# Clinical and Radiological Results of Hemiarthroplasty and Total Shoulder Arthroplasty for Primary Avascular Necrosis of the Humeral Head in Patients Less Than 60 Years Old

**DOI:** 10.3390/jcm10143081

**Published:** 2021-07-12

**Authors:** Anthony Hervé, Mickael Chelli, Pascal Boileau, Gilles Walch, Luc Favard, Christophe Levigne, François Sirveaux, Philippe Clavert, Nicolas Bonnevialle, Philippe Collin

**Affiliations:** 1 Rennes Ortho Sport, Polyclinique St Laurent Rennes, 35000 Rennes, France; 2 Institut de Chirurgie Réparatrice Locomoteur et Sports, 06004 Nice, France; mickael.chelli@gmail.com (M.C.); boileau.pascal@wanadoo.fr (P.B.); 3 Clinique Santy, 69008 Lyon, France; gilleswalch15@gmail.com; 4 CHU Tours, 37000 Tours, France; luc.favard@wanadoo.fr; 5 Clinique du Parc, 69006 Lyon, France; dr.levigne@cliniqueduparclyon.fr; 6 CHU Nancy, 54000 Nancy, France; francois.sirveaux@wanadoo.fr; 7 CHU Strasbourg, 67200 Strasbourg, France; philippe.clavert@chru-strasbourg.fr; 8 CHU Toulouse Pierre Paul Ricquet, 31300 Tolouse, France; nicolasbonnevialle@wanadoo.fr; 9 Institut Locomoteur de l’Ouest, CHP St Grégoire, 35760 Saint Grégoire, France; docphcollin@gmail.com

**Keywords:** shoulder arthroplasty, glenohumeral osteoarthritis, avascular necrosis of the humeral head, hemi arthroplasty, total shoulder arthroplasty, young patients

## Abstract

Background: Total shoulder arthroplasty (TSA) and hemiarthroplasty (HA) have shown good clinical outcomes in primary avascular necrosis of the humeral head (PANHH) both in short and long terms. The purpose of this study was to assess the complications, the clinical and radiological outcomes of shoulder arthroplasty in young patients with PANHH. Methods: One hundred and twenty-seven patients aged under 60 years old and suffering from PANHH were operated with arthroplasty. Patients were assessed clinically and radiographically before surgery with a minimum of 2 years of follow up (FU). Results: HA was performed on 108 patients (85%). Two patients were revised for painful glenoid wear after 2 and 4 years. TSA was performed on 19 patients (15%). Five TSA had to be revised for glenoid loosening (*n* = 4) or instability (*n* = 1). Revision rate was 26% with TSA and 2% with HA. There were no significant differences between HA and TSA in terms of clinical outcomes. Conclusions: With a mean FU of 8 years, HA and TSA improved clinical outcomes of patients with PANHH. HA revisions for painful glenoid wear were rare (2%). The revision rate was excessively high with TSA (26%).

## 1. Introduction

Primary avascular necrosis of the humeral head (PANHH) is the result of the necrosis of the bone tissue and bone marrow of the humeral head. It often affects patients in their 4th or 5th decade. While many etiologies are linked to corticosteroid therapy or alcohol abuse, most of the time no causes are identified. Several studies have investigated the results of shoulder arthroplasty for PANHH. Total shoulder arthroplasty (TSA) [1,2,3,4,5] and hemiarthroplasty (HA) [1,6,7,8,9,10,11] have shown satisfactory clinical outcomes in short-, mid- and long-term follow-up (FU). It has been well demonstrated that post-traumatic necrosis resulted in inferior outcomes than PANHH [1,12,13]. Only two studies [1,4] have compared the results of HA and TSA but gathered primary and post-traumatic AVHH [1] or the two groups were not comparative in terms of age and age related factor [4]. Therefore, our aim was to assess the complications, the clinical and radiological outcomes of shoulder arthroplasty for PANHH in patients aged 60 years old or younger at the time of the surgery. The hypotheses were that HA and TSA would (1) both improve clinical outcomes during the FU midterm (2) but would differ with TSA having a higher rate of complication than HA.

## 2. Materials and Methods

### 2.1. Patients

A multicenter retrospective study among 9 centers was conducted. Inclusion criteria were patients suffering from PANHH operated with total shoulder arthroplasty (TSA) or hemiarthroplasty (HA), aged 60 years old or under at the time of the surgery. Exclusion criteria were post-traumatic avascular necrosis, and less than 2 years between surgery and last follow-up (FU) for clinical and radiological analysis.

One hundred and twenty-seven patients were operated between 1991 and 2015 with a mean age of 46 years old (SD 10, range 19–60): 108 HA and 19 TSA. The etiologies of PANHH were Churg and Strauss disease (*n* = 2), corticotherapy for Hodgkin lymphoma (*n* = 2), drepanocytosis (*n* = 1) and post-radiotherapy (*n* = 1). For the other 121 patients (95%), no specific etiologies were found, and osteonecrosis was therefore classified as idiopathic. Five patients had undergone conservative treatment prior to arthroplasty: micro-fractures (*n* = 3), arthroscopic suprasupinatus repair (*n* = 1) and acromioplasty (*n* = 1).

We evaluated clinical outcomes with passive and active range of motion using the Constant score [14] and Subjective shoulder value [15] (SSV). Radiographic evaluation consisted of true anteroposterior radiographs of the gleno-humeral joint using a standardized protocol during the preoperative evaluation and the last follow-up. The osteonecrosis severity was assessed with Ficat’s [16] classification modified by Cruess [17] (Table 1).

Radiographs were evaluated by a senior and a resident orthopedic surgeon. Pre-operative radiographs were missing for 6 patients. Radiolucent lines (RLL) around the humeral stem and the glenoid component (of TSA) were assessed with Mole score [18]. All patients provided informed consent for their participation in this study, which had been approved by the institutional review board.

### 2.2. Operative Technique

The operative technique was performed in a beach-chair position under general anesthesia with an inter-scalene block. The surgical approach was almost exclusively deltopectoral and anterosuperior in 2 cases (2%). Tenodesis and subscapularis repair were systematic. For 2 patients (2 HA), the cuff tear was also repaired while undergoing the arthroplasty. The type of arthroplasty was left to the operator’s choice and to the severity of the osteoarthritis.

### 2.3. Hemiarthroplasty Group

Hemiarthroplasty was performed on 108 patients (85%). On pre-operative radiographs, Ficat and Cruess classification stages were in 12 times stage 2, in 36 times stage 3, in 37 times stage 4 and in 21 times stage 5. Two pre-operative radiographs were missing (1, 8%). Hemi-metal implants were implanted 67 times (62%), 6 (5%) hemi pyrocarbone, 19 (18%) pyrocarbone interposition (PI) sphere and 16 (15%) resurfacing. Overall a stem was used in 67% of times. Hemi-metal implants included 63 Aequalis Anatomic (Tornier SAS-Wright Medical, Bloomington, MN, USA). Among them 6 were uncemented. Otherwise 4 uncemented Ascend flex anatomic were used (Tornier SAS-Wright Medical). Hemi-pyrocarbone implants were uncemented Ascend Flex anatomic stems (Tornier SAS-Wright Medical). PI spheres corresponded to Inspyre implant (Tornier SAS-Wright Medical). Resurfacing and stemless implants consisted of 6 Aequalis Resurfacing Head (Tornier SAS-Wright Medical), 4 Copeland (Biomet, Inc., Warsaw, IN, USA), 2 Global Cap Resurfacing (DePuy Orthopaedics, Warsaw, IN, USA), 2 TESS (Total Evolutive Shoulder System) (Biomet, Inc., Warsaw, IN, USA), 1 HemiCAP-Arthrosurface ^®^ system (Arthrosurface, Inc., Franklin, MA, USA) and 1 Affinis stemless (Mathys, Bettlach, Switzerland).

### 2.4. Total Anatomic Arthroplasty

Total anatomic arthroplasty was performed on 19 patients (15%). On pre-operative radiographs, Ficat and Cruess classification was in 1 time stage 3 and in 14 times stage 5. Four pre-operative radiographs were missing. On the glenoid side, 3 (16%) metal-backed and 16 (84%) keeled full polyethylene (PE) components were used. Humeral stems consisted of Aequalis anatomic for 17 (Tornier SAS-Wright Medical) and Ascend flex stems (Tornier SAS-Wright Medical) for 2. The stem was cemented for 78% of the procedures.

### 2.5. Statistical Analysis

Data collection and statistical analysis were investigated with the free online software EasyMedStat (www.easymedstat.com; Neuilly-Sur-Seine; France). Continuous data were expressed as mean (standard deviation, minimum–maximum) and categorical data were given as absolute and relative frequencies (%). To compare differences between preoperative and last FU data, the Student t-test for paired data or the Wilcoxon signed-rank test were used accordingly. Survival rate without a revision surgery and its 5% pointwise confidence intervals were estimated with the method of Kaplan Meier. The significance level was set at *p* < 0.05.

## 3. Results

### 3.1. Postoperative Complications

Thirteen patients (9%) suffered from postoperative complications: 6 (6%) in the HA group and 7 (37%) in the TSA group (OR = 9.9; 95% CI = (2.9; 34.4); *p* < 0.001). In the HA group, 2 painful glenoid wear complications were observed as well as 1 humeral shaft fracture, 1 coagulase-negative Staphylococcus infection, 1 cuff tear involving supraspinatus and subscapularis, and 1 ulnar nerve palsy which recovered in 3 months. In the TSA group, one patient had an immediate posterior dislocation with a metal-back glenoid.

### 3.2. Reoperations

Three HA were reoperated without a humeral stem revision: One patient suffered from a humerus shaft fracture after a trauma and was treated by plating. Another patient had a massive cuff tear type A [19] after a severe trauma in a motor bike accident and was repaired with an open approach. The last patient had an arthroscopic biopsy due to acute pain which appeared one year after the surgery. Bacterial cultures were negatives. During the last FU (116 months after), this last patient’s range of motion was excellent. Nevertheless, Constant score was 61, especially because the patient was still in pain. None of the TSA that needed to be reoperated had to undergo a revision of prosthetic components.

### 3.3. Revisions

**Figure 1:**
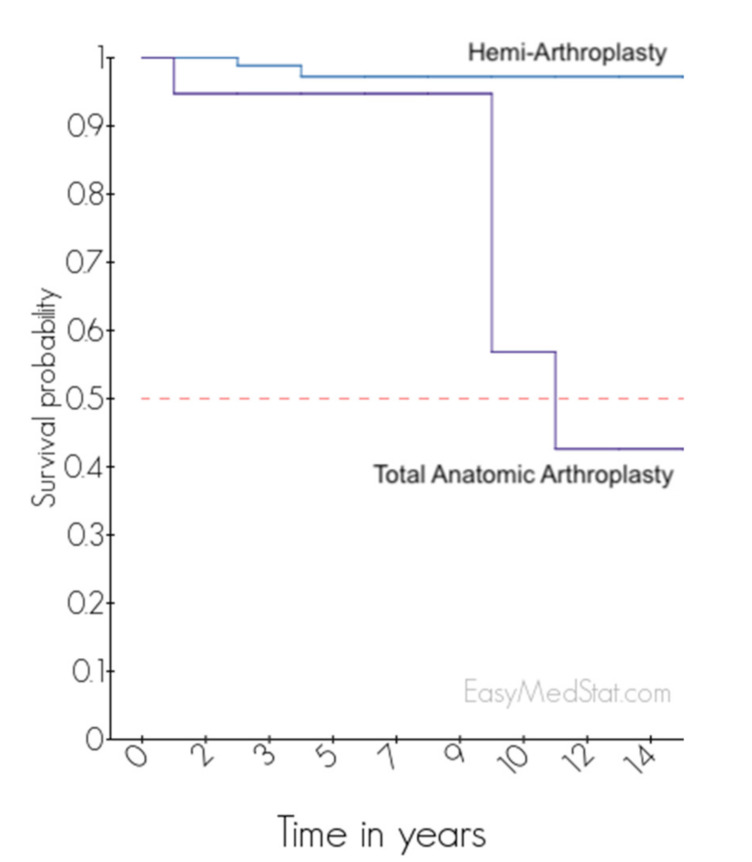
Survival curve of HA and TSA after PANHH.

Revision surgery was required for 7 patients (6%) including 2 HA (2%) and 5 TSA (26%) (OR = 20; (95% CI = 4–116); *p* < 0.001). Survival rate without revision at 5 years was 97% (89–99%) for HA and 97% (89–99%) for TSA. At 10 years, survival rate without revision was 95% (68–99%) for HA and 57% (19–82%) for TSA (*p* < 0.001) (Figure 1).

**Figure 2:**
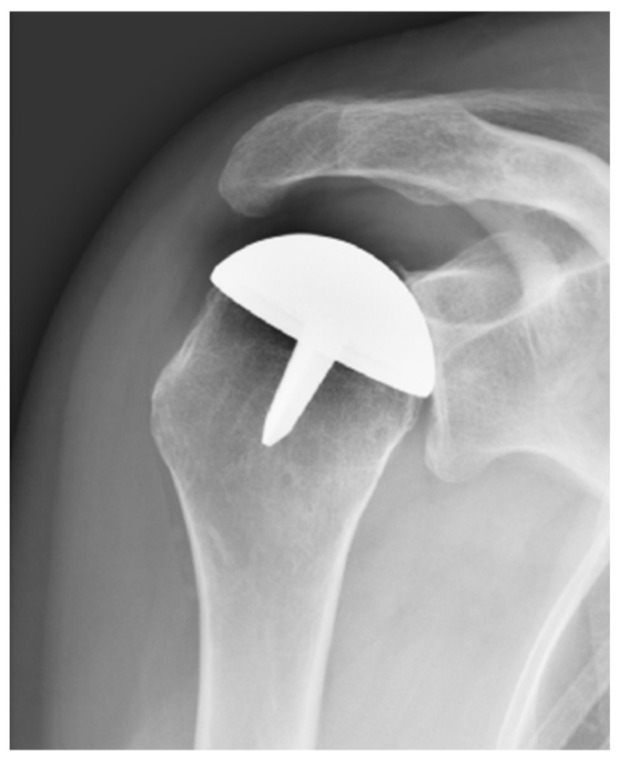
Resurfacing complicated by glenoid wear at 49 months of FU.

In the HA group, 2 were revised for glenoid wear. The stage of Ficat’s classification in pre-operative radiographs was stage 3. The first one was a resurfacing HA (Figure 2) complicated with painful glenoid wear at 49 months and reoperated with a pyrocarbone HA. During the final FU, 2 years after the revision surgery, the Constant score was 55 and SSV 80%.

**Figure 3:**
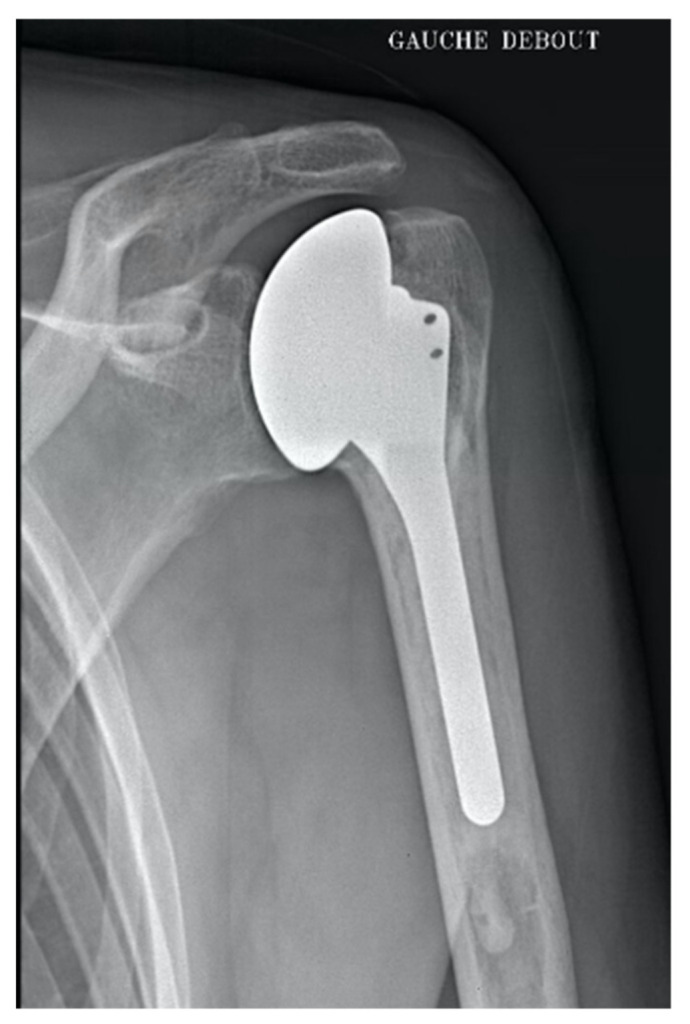
HA metal complicated by glenoid wear at 25 months of FU.

The second case was a metal HA where glenoid wear occurred at 25 months of FU (Figure 3). The humeral stem was unchanged and a cemented full PE glenoid was implanted. During the final FU, 3 years after the revision surgery, the Constant score was 57 and SSV 70%.

**Figure 4:**
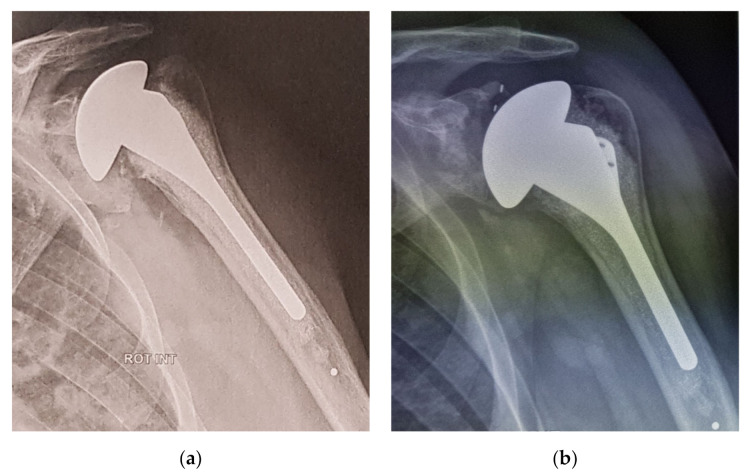
(**a**) TSA complication due to glenoid loosening at 9 years FU and (**b**) reoperated using an HA.

In the TSA group, one patient who had a posterior instability underwent a full-PE implant but due to a glenoid loosening 5 years later, it lead to a definitive explanation of both humeral and glenoid components with a poor functional result (CS = 15). Three patients with glenoid loosening were reoperated using HA (Figure 4).

### 3.4. Clinical Outcomes

For clinical and radiological analysis, patients who had their FU within 2 years, and those who sustained a revision of their primary arthroplasty were excluded, leaving 92 patients: 83 HA with a mean FU of 8.2 years (SD: 5.2, range: 2–26) and 9 TSA with a mean FU of 8.8 years (SD: 5.6, range: 2–18) (*p* = 0.84). At the last FU, both HA and TSA were significantly improved regarding CS, and SSV (*p* < 0.01) with a mean CS of 76 for HA and 71 for TSA (*p* = 0.35). Details and range of motion are provided in Table 2.

### 3.5. Radiological Analysis

At last FU, the mean humeral RLL score was 0.02 (range 0–1) for HA and 2.0 (range 0–6) for TSA (*p* = 0.002). In the HA group, there was no significant trend between glenoid bone wear and the pre-operative stage of Ficat and Cruess classification (*p* > 0.05). In the TSA group, the mean glenoid RLL score was 5.2 (range 0–18) with 2 migrated glenoid implants, considered as loosened but no revision surgery had been done (CS = 49 and 67). After 7 to 10 years postoperatively, glenoid loosening was observed in 6 patients who had not received revision surgery. There was no significant trend between glenoid loosening and the pre-operative stage of Ficat and Cruess classification (*p* > 0.05).

### 3.6. Comparison between Different Hemi-Arthroplasties

There were no significant differences between HA regarding postoperative complications, revision surgery and clinical outcomes (Table 3).

## 4. Discussion

The purpose of our study was to evaluate clinically and radiographically the outcomes of shoulder arthroplasty in young patients with PANHH. Both HA and TSA improved the clinical function of affected patients significantly after 8.2 years (mean FU). Revision surgeries for glenoid wear were low (2%). Complications after TSA were excessively high with 32% exhibiting glenoid loosening and 26% receiving revision surgery.

Mansat et al. [2] reported outcomes on 19 HA with a mean FU of 7 years. Mean Constant score (58 points) was significantly improved at last FU. Post-irradiation PANHH yielded the worst results. At long term, with a mean FU set at 12 years, Smith et al. [6] confirmed in 31 HA that mean motion range had still improved significantly (*p* < 0.001).

In our study, two hemi-metal implants (2%) had revision surgery for glenoid wear at 2.4 and 4.1 years postoperatively, with pre-operative Ficat classification at stage 3. Mansat et al. [2] related painful glenoid wear developed in 2 of the 14 HA (14%) at 6.2 and 9.6 years of FU. Only one patient with low Constant score (33 points) had revision surgery. At long term, Smith et al. [6] noted 14 glenoid erosions out of 23 shoulders (61%), but only 2 patients had revision surgery for TSA (7%). The estimated survival rate for HA in their study was 100% after 5 years and 92% after 10 and 15 years.

In our study, survival rate without revision surgery was 97% (89–99%) and 95% (68–99%) at 5 and 10 years. No significant differences between different types of HA, regarding postoperative complication, revision surgery or clinical outcomes were observed. Nevertheless, anatomic cemented stems with metal head (Aequalis, Tornier SAS-Wright Medical) were the device which had the longest follow up and excellent treatment outcomes.

In Herschel et al. [20] study, valgus position of the prosthetic humeral and glenoid cysts were identified as risk factors for glenoid erosion after HA. The size of the humeral head component was not correlated with glenoid erosion in the study of Al-Hadithy et al. [21].

TSA gave excellent results at short and middle term but it exposed patients to glenoid implant loosening.

In our study, glenoid loosening occurred in 6 of 19 TSA (31.6%) between 7 and 10 years FU but this cohort was small. Four TSA had been reoperated. Two glenoids components considered as loosened did not undergo a new surgery (CS = 49 and 67).

Schoch et al. [4] followed 71 TSA after PANHH with a mean follow up of 7.7 years. Pain and range of motion were significantly improved. Among them, 11 (15%) underwent reoperation at a mean time of 4.4 years (range, 0.6–11 years) after index arthroplasty. Four patients (5%) needed to be reoperated for aseptic glenoid loosening. 

In a prospective study, Parch et al. [3] prospectively evaluated 13 TSA at a mean follow-up of 30.2 months (range, 14–49 months). Shoulder function assessed by the Constant score improved from 18 (adjusted score, 24%) to 51 (adjusted score, 69%; *p* < 0.001). They observed that patients younger than 65 years obtained lower adjusted Constant scores (mean, 58%; *n* = 7) than patients older (mean, 82%; *n* = 6; *r*_s_ = −0.73, *p* = 0.02). During follow up, the patient with the lowest adjusted Constant scores was the one with progressive glenoid erosion preoperatively.

Relatively few studies compared the outcomes between HA and TSA for PANHH in the literature. Recently, a study by Ristow et al. [5] assessed 10 TSA and 19 HA and showed no significant differences in clinical outcomes with a mean follow-up of 3.9 years (range, 1–8.5 years). Mean age at surgery was 49.2 years (range, 16–77 years). It demonstrated a trend of better outcome scores with TSA but without statistical significance. Traumatic cases concerned 20% of their patients which impacted the results.

Feeley et al. [1] compared 26 HA vs. 17 TSA with 4.8 years of FU. TSA was associated with lower ASES score and decreased forward flexion compared to hemiarthroplasty (*p* < 0.05). There were significantly more reoperations in the TSA group (22%) among which 4 exhibited glenoid loosening. Schoch et al. [4] compared 67 HA vs. 71 TSA with a mean FU of 9.3 years. At the time of final follow-up, active elevation was significantly higher in the HA group (*p* = 0.04).

In our study, despite a shorter follow up with HA, 2 HA had revision surgery for glenoid wear with a mean follow up of 11.9 years. Twenty years later, the percentage of reoperation-free patients was calculated to be 87%. Fifteen percent of TSA had revision surgery with a mean time of 4.4 years at index surgery. Four of eleven patients were reoperated for aseptic glenoid loosening. Reoperation-free survival rate was calculated to be 79% (CI, 67–92). 

Our study has inherent limitations due to its retrospective and multicentric design. It mixed different kinds of hemiarthroplasties with a heterogeneous follow-up. Moreover, the cohort of TSA was smaller with a smaller FU than HA. Nevertheless, it analyses one of the longest FU in the literature about shoulder arthroplasty for PANHH. There were no statistical differences between clinical outcomes and post-operative complications with the stage of the osteonecrosis. Glenoid wear occurred rarely after HA. TSA seemed to be complicated by glenoid loosening more. Humeral metal-head implants gave excellent results and are still a good option for HA.

## 5. Conclusions

With a mean follow-up of 8 years, HA and TSA improved significantly clinical outcomes in patients with PANHH. Revision surgeries of HA for painful glenoid wear were rare (2%), but the revision rate for glenoid loosening was high with TSA (26%).

## Figures and Tables

**Table 1:** Pre-operative radiographs assessed by Ficat’s classification modified by Cruess.

	** 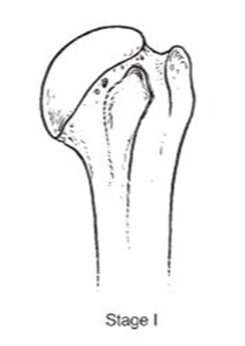 **	** 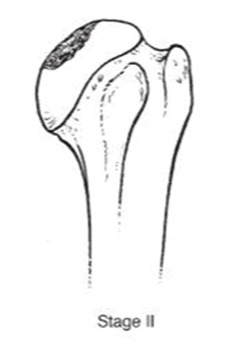 **	** 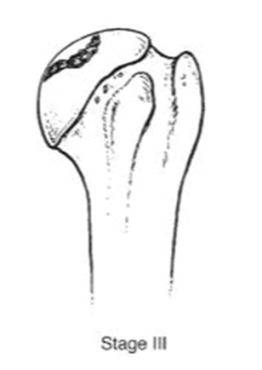 **	** 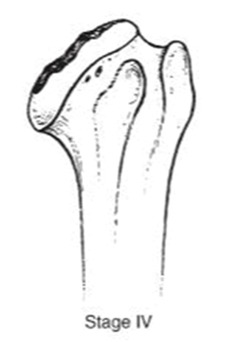 **	** 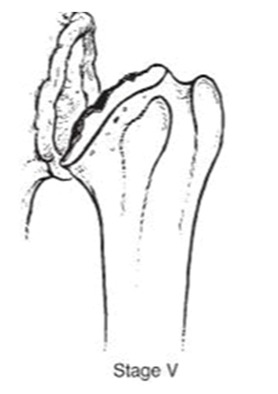 **
***n* =**	0	4 (3.3%)	38 (31.4%)	40 (33%)	39 (3.3%)

**Table 2:** Clinical outcomes in hemi-arthroplasty and total anatomic arthroplasty surgeries.

	Forward Elevation (°)	External Rotation (°)	Internal Rotation (/10)	CS (/100)	SSV (%)
**Hemi-arthroplasty (*n* = 83)**					
**Preoperative**	105 ± 37 (30–180)	22 ± 18 (−10–70)	4.7 ± 2.5 (0–10)	37 ± 14 (10–71)	39 ±17 (5–70)
**Last FU**	154 ± 23 (90–180)	39 ± 19 (−10–85)	7.1 ± 1.9 (2–10)	76 ± 11 (50–96)	87 ± 13 (60–100)
***p*** **-Value ^a^**	<0.001 *	<0.001 *	<0.001 *	<0.001 *	<0.001 *
**Total Anatomic Arthroplasty (*n* = 9)**					
**Preoperative**	112 ± 29 (80–160)	14 ± 19 (−10–45)	4.2 ± 2.9 (0–10)	43 ± 11 (28–55)	33 ± 14 (20–50)
**Last FU**	142 ± 33 (90–170)	39 ± 18 (10–60)	6.2 ± 2.9 (2–10)	71 ± 20 (43–94)	73 ± 6 (70–80)
***p*** **-Value ^a^**	0.055	<0.01 *	0.022 *	<0.01 *	<0.001 *

^a^ *p*-Value for the difference between preoperative and last FU (Wilcoxon signed-rank test * *p* < 0.05).

**Table 3:** Comparison of complications, revision surgery and clinical outcomes among different hemi-arthroplasties.

	Metal-Head (*n* = 67)	Pyrocarbon (*n* = 6)	Resurfacing (*n* = 16)	Interposition Sphere (*n* = 19)	*p*-Value
**Postoperative Complication (%)**	6%	0%	6%	5%	1
**Revision surgery (%)**	1%	0%	6%	0%	0.40
	**Metal-Head (*n* = 47)**	**Pyrocarbon (*n* = 5)**	**Resurfacing (*n* = 13)**	**Interposition Sphere (*n* = 18)**	***p*-Value**
**Forward Elevation (°)**	155 ± 22 (90–180)	148 ± 27 (110–170)	149 ± 27 (100–180)	154 ± 23 (110–180)	0.98
**External Rotation (°)**	35 ± 18 (−10–70)	53 ± 15 (45–80)	44 ± 16 (20–70)	42 ± 21 (−10–85)	0.09
**Internal Rotation (/10)**	6.6 ± 2.0 (2–10)	8.4 ± 1.7 (6–10)	6.8 ± 2.4 (4–10)	7.7 ± 1.5 (4–10)	0.14
**Constant Score (/100)**	73 ± 14 (30–91)	75 ± 10 (61–89)	75 ± 13 (54–96)	78 ± 10 (61–95)	0.79
**SSV (%)**	82 ± 21 (40–100)	86 ± 9 (80–100)	82 ± 11 (60–100)	89 ± 12 (65–100)	0.46

## Data Availability

The data can be found at www.easymedstat.com.

## References

[1] Feeley B.T., Fealy S., Dines D.M., Warren R.F., Craig E.V. (2008). Hemiarthroplasty and Total Shoulder Arthroplasty for Avascular Necrosis of the Humeral Head. J. Shoulder Elb. Surg..

[2] Mansat P., Huser L., Mansat M., Bellumore Y., Rongières M., Bonnevialle P. (2005). Shoulder Arthroplasty for Atraumatic Avascular Necrosis of the Humeral Head: Nineteen Shoulders Followed up for a Mean of Seven Years. J. Shoulder Elb. Surg..

[3] Parsch D., Lehner B., Loew M. (2003). Shoulder Arthroplasty in Nontraumatic Osteonecrosis of the Humeral Head. J. Shoulder Elb. Surg..

[4] Schoch B.S., Barlow J.D., Schleck C., Cofield R.H., Sperling J.W. (2016). Shoulder Arthroplasty for Atraumatic Osteonecrosis of the Humeral Head. J. Shoulder Elb. Surg..

[5] Ristow J.J., Ellison C.M., Mickschl D.J., Berg K.C., Haidet K.C., Gray J.R., Grindel S.I. (2019). Outcomes of Shoulder Replacement in Humeral Head Avascular Necrosis. J. Shoulder Elb. Surg..

[6] Smith R.G., Sperling J.W., Cofield R.H., Hattrup S.J., Schleck C.D. (2008). Shoulder Hemiarthroplasty for Steroid-Associated Osteonecrosis. J. Shoulder Elb. Surg..

[7] Raiss P., Kasten P., Baumann F., Moser M., Rickert M., Loew M. (2009). Treatment of Osteonecrosis of the Humeral Head with Cementless Surface Replacement Arthroplasty. J. Bone Jt. Surg. Am..

[8] Ohl X., Nérot C., Saddiki R., Dehoux E. (2010). Shoulder Hemi Arthroplasty Radiological and Clinical Outcomes at More than Two Years Follow-Up. Orthop. Traumatol. Surg. Res..

[9] Uribe J.W., Botto-van Bemden A. (2009). Partial Humeral Head Resurfacing for Osteonecrosis. J. Shoulder Elb. Surg..

[10] Sweet S.J., Takara T., Ho L., Tibone J.E. (2015). Primary Partial Humeral Head Resurfacing: Outcomes with the HemiCAP Implant. Am. J. Sports Med..

[11] Soudy K., Szymanski C., Lalanne C., Bourgault C., Thiounn A., Cotten A., Maynou C. (2017). Results and Limitations of Humeral Head Resurfacing: 105 Cases at a Mean Follow-up of 5 Years. Orthop. Traumatol. Surg. Res..

[12] Sowa B., Thierjung H., Bülhoff M., Loew M., Zeifang F., Bruckner T., Raiss P. (2017). Functional Results of Hemi- and Total Shoulder Arthroplasty According to Diagnosis and Patient Age at Surgery. Acta Orthop..

[13] Orfaly R.M., Rockwood C.A., Esenyel C.Z., Wirth M.A. (2007). Shoulder Arthroplasty in Cases with Avascular Necrosis of the Humeral Head. J. Shoulder Elb. Surg..

[14] Constant C.R., Murley A.H. (1994). A Clinical Method of Functional Assessment of the Shoulder. Clin. Orthop. Relat. Res..

[15] Gerber C., Vinh T.S., Hertel R., Hess C.W. (1992). Latissimus Dorsi Transfer for the Treatment of Massive Tears of the Rotator Cuff. A Preliminary Report. Clin. Orthop. Relat. Res..

[16] Ficat P., Arlet J. (1973). Pre-radiologic stage of femur head osteonecrosis: Diagnostic and therapeutic possibilities. Rev. Chir. Orthop. Reparatrice Appar. Mot..

[17] Cruess R.L. (1976). Steroid-Induced Avascular Necrosis of the Head of the Humerus. Natural History and Management. J. Bone Jt. Surg. Br..

[18] Molé D., Roche O., Riand N., Lévigne C., Walch G. (1999). Cemented Glenoid Component: Results in Osteoarthritis and Rheumatoid Arthritis. Shoulder Arthroplasty.

[19] Collin P., Matsumura N., Lädermann A., Denard P.J., Walch G. (2014). Relationship between Massive Chronic Rotator Cuff Tear Pattern and Loss of Active Shoulder Range of Motion. J. Shoulder Elb. Surg..

[20] Herschel R., Wieser K., Morrey M.E., Ramos C.H., Gerber C., Meyer D.C. (2017). Risk Factors for Glenoid Erosion in Patients with Shoulder Hemiarthroplasty: An Analysis of 118 Cases. J. Shoulder Elb. Surg..

[21] Al-Hadithy N., Domos P., Sewell M.D., Naleem A., Papanna M.C., Pandit R. (2012). Cementless Surface Replacement Arthroplasty of the Shoulder for Osteoarthritis: Results of Fifty Mark III Copeland Prosthesis from an Independent Center with Four-Year Mean Follow-Up. J. Shoulder Elb. Surg..

